# Identification of Pre-Diagnostic Metabolic Patterns for Glioma Using Subset Analysis of Matched Repeated Time Points

**DOI:** 10.3390/cancers12113349

**Published:** 2020-11-12

**Authors:** Pär Jonsson, Henrik Antti, Florentin Späth, Beatrice Melin, Benny Björkblom

**Affiliations:** 1 Department of Chemistry, Umeå University, SE-901 87 Umeå, Sweden; par.jonsson@umu.se (P.J.); henrik.antti@umu.se (H.A.); 2 Department of Radiation Sciences, Oncology, Umeå University, SE-901 87 Umeå, Sweden; florentin.spaeth@umu.se (F.S.); beatrice.melin@umu.se (B.M.)

**Keywords:** brain tumor, metabolite, metabolic marker pattern, multivariate analysis, blood-based, antioxidant

## Abstract

**Simple Summary:**

Reprogramming of cellular metabolism is a major hallmark of cancer cells, and play an important role in tumor initiation and progression. The aim of our study is to discover circulating early metabolic markers of brain tumors, as discovery and development of reliable predictive molecular markers are needed for precision oncology applications. We use a study design tailored to minimize confounding factors and a novel machine learning and visualization approach (SMART) to identify a panel of 15 interlinked metabolites related to glioma development. The presented SMART strategy facilitates early molecular marker discovery and can be used for many types of molecular data.

**Abstract:**

Here, we present a strategy for early molecular marker pattern detection—Subset analysis of Matched Repeated Time points (SMART)—used in a mass-spectrometry-based metabolomics study of repeated blood samples from future glioma patients and their matched controls. The outcome from SMART is a predictive time span when disease-related changes are detectable, defined by time to diagnosis and time between longitudinal sampling, and visualization of molecular marker patterns related to future disease. For glioma, we detect significant changes in metabolite levels as early as eight years before diagnosis, with longitudinal follow up within seven years. Elevated blood plasma levels of myo-inositol, cysteine, N-acetylglucosamine, creatinine, glycine, proline, erythronic-, 4-hydroxyphenylacetic-, uric-, and aceturic acid were particularly evident in glioma cases. We use data simulation to ensure non-random events and a separate data set for biomarker validation. The latent biomarker, consisting of 15 interlinked and significantly altered metabolites, shows a strong correlation to oxidative metabolism, glutathione biosynthesis and monosaccharide metabolism, linked to known early events in tumor development. This study highlights the benefits of progression pattern analysis and provide a tool for the discovery of early markers of disease.

## 1. Introduction

Circulating biomarkers are increasingly utilized in the advance towards molecular medicine. Blood-based biomarkers are needed for patient stratification, early detection of disease for personalized screening in risk groups, and for the development of new therapies for patients with poor prognosis [1,2]. Biomarker development is a multistep and iterative process, where screening blood samples from human population biobanks have received increasing interest. ‘Multi-omics’ approaches are commonly applied, which implies looking for disease-related systematic differences or trends in high-dimensional data. Identifying these differences can be challenging in the presence of other systematic variation sources and random noise, complications that dilute the variation of interest. Typically, this dilution is addressed by increasing the power of cross-sectional studies to achieve statistical significance. However, this approach requires large sample sizes that are often difficult to obtain and technically challenging to analyze, and with lower effect sizes that can have limited clinical value. Uncontrolled extraneous variables and confounders may also require post-correction of the data to establish biomarker significance, a sub-optimal approach in terms of both sensitivity and reliability of biomarkers [3]. A valid alternative is the use of tightly controlled smaller sample studies that include repeated time points and thoroughly matched case-control sets. Repeated or longitudinal sampling of both cases and controls enables between-within subject normalization. Subtracting the baseline sample from the repeated sample gives the progression pattern, normalizing individual differences and emphasize change over time. By comparing the progression pattern with a tightly matched control, one will further reduce extraneous influences associated with the passage of time and sample storage, and detect disease-related variation. This approach minimizes confounding factors related to individual differences and passage of time, allowing for higher sensitivity for detection of disease-related markers. In the subsequent data analysis, sample dependency can be used to optimize the information recovery. Until recently, analysis of dependent samples has not been straightforward in multivariate statistical analysis. With the effect projection approach based on orthogonal projections to latent structures (OPLS-EP) [4], information recovery from dependent samples is now possible within the OPLS framework, including cross-validation, model statistics, interpretation and predictions.

Motivated by the need to discover early molecular markers and risk factors for brain tumors, we describe here a data acquisition, projection and visualization approach: Subset analysis of Matched Repeated Time points (SMART), used for the extraction of variables for molecular marker pattern detection in prospective biobank samples. We investigate the value of tightly controlled sample studies that include repeated time points and thoroughly matched case-control sets. We exemplify our strategy in two independent sets of plasma samples, collected and stored under identical conditions, from pre-diagnostic glioma cases and stringently matched controls. We find that the use of repeated samples result in significant models, separating future cases from controls. Using dependent samples, SMART enable us to define a molecular marker pattern related to the disease and a pre-diagnostic period when the pattern starts to appear, without supervised post correction of the data. We use a second set of plasma samples to verify the time point for disease detection and the identified biomarker pattern. This proof-of-concept study demonstrate the benefits of progression pattern analysis combined with multivariate statistical analysis, and indicate that metabolic changes for glioma start to emerge many years before diagnosis.

## 2. Results

### 2.1. Progression Pattern Analysis

For metabolic marker detection, we used EDTA-plasma samples from a prospective population-based biobank. The sample set consisted of tightly matched case-control samples collected longitudinally with repeated pre-diagnostic time points (Table S1). We performed metabolite measurements by use of gas chromatography mass spectrometry (GC–MS) and constrained run order, keeping matched samples together in the analysis process and only randomizing between and within the matched sample groups [4]. For the generated data, we analyzed case-control differences by independent multivariate analysis using OPLS-DA [5] and dependent multivariate analysis using OPLS-EP [4]. Calculation of the disease progression pattern for the repeated time point samples, normalizing individual differences for both future cases and controls, was done by subtracting baseline data from the repeated time point data closer to diagnosis (Equation (1)), followed by calculating the differences in progression between matched future cases and controls (Equation (2)).

X_progress_ = X_repeated_ − X_baseline_ (1)

X_disease progress_ = X_progress (case)_ − X_progress (control)_ (2)

A summary of the modeling results is presented in Table 1. Our modeling of progression pattern shows that a statistically significant model can be obtained using case-control progression pattern analyzed by dependent sample analysis, i.e., OPLS-EP (*P*-value, analysis of variance of cross-validated residuals (CV-ANOVA) = 0.026). This indicates that a progression pattern related to future glioma exists and that there are benefits of retaining sample dependency, and minimizing confounding variation related to individual differences and passage of time between sampling.

### 2.2. Outline of the SMART Procedure

**Figure 1:**
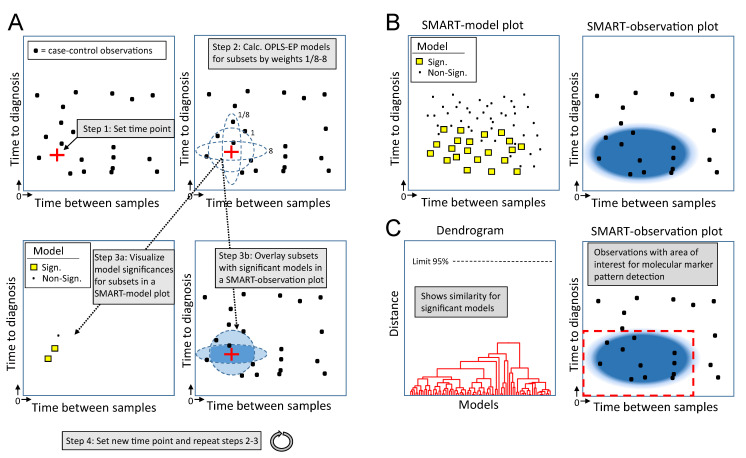
Schematic outline of the Subset analysis of Matched Repeated Time points (SMART) procedure. (**A**) Each case-control observation, containing information for disease progression (Equation (2)), is plotted in a scatter plot according to time to diagnosis and time between repeated sampling. Step 1: The algorithm selects a random time point in the scatter plot. Step 2: Weights are used to scan the area surrounding the selected time point, and an OPLS-EP model is calculated for every subset of case-control observations closest in time (distance) to the selected time point. For simplicity, this illustration shows only three weight used for scanning (ellipses 1/8, 1 and 8), generating three subsets and three corresponding OPLS-EP models. Step 3a: Significance of the calculated OPLS-EP models is visualized in a new scatter plot, i.e., a SMART-model plot, where a yellow square (□) marks a significant, and a black dot (∙) a non-significant OPLS-EP model. In this illustration, two out of three subsets generated significant models. The positions of OPLS-EP model markers (□ or ∙) in the scatter plot are determined by the mean values for the case-control observations included in the analyzed subset. Step 3b: In parallel, a SMART-observation plot is generated, which shows included subsets from which significant models were obtained. This is visualized by creating a 2D density plot where the color density correlates to the number of overlaid subsets generating significant OPLS-EP models only. Step 4: A new time point is selected and previous steps are repeated until the whole matrix has been scanned. (**B**) All model significances are summarized in a final SMART-model plot, showing the distribution of significant and non-significant OPLS-EP models. In parallel, a SMART-observation plot is summarized, showing all case-control observations and the distribution pattern of corresponding subsets with significant OPLS-EP models. (**C**) Unsupervised hierarchical cluster analysis is performed to show the similarity of significant OPLS-EP models. In this illustration, only one cluster of significant models is observed in the generated dendrogram. This cluster corresponds to a region of interest, with a predictive value for molecular marker pattern detection, in the SMART-observation plot (red dashed line square).

We developed SMART to systematically and without bias dig deeper into the data and find out what the significant metabolic patterns are and when they start to appear. Using both baseline and repeated samples, we investigated two factors: (i) time between repeated samples, i.e., the time span between collection of the baseline and the repeated sample, and (ii) time to diagnosis, i.e., the time span between disease diagnosis and the collection of the repeated sample. The obvious hypothesis being that metabolic changes related to glioma can be detected in plasma, at some time point before diagnosis. A constrain being that baseline and repeated samples collected very far apart might not serve as proper references for within-subject normalization, due to altered metabolism in relation to, e.g., aging and possible change of lifestyle. In the matrix, or scatter plot defined by time to diagnosis and time between repeated samples, the SMART procedure was used to define a region of interest where the measured variables show a predictive value. The SMART procedure defines the region of interest by calculation of OPLS-EP models for subsets of the data and visualization of the subset models and their significance. In Figure 1A, we outline the SMART procedure in a few schematic step-by step illustrations so that the readers can better understand how the algorithm works and interpret the generated results. More detailed information and parameters used for the SMART procedure is described in the materials and methods section and in the Figure 1 legend. The final output from the SMART procedure is a SMART-model plot, showing the distribution of significant and non-significant OPLS-EP models, and a SMART-observation plot, showing all case-control observations and distribution of corresponding subsets with significant OPLS-EP models (Figure 1B). Based on this data, we can define a region of interest with variables showing a predictive value for molecular marker detection, visually seen as a cluster of significant models and contributing subsets. To define this region in an unbiased way, we applied unsupervised hierarchical clustering analysis, showing similarities between significant models. The created dendrogram displays clustering of metabolic patterns with the same or very similar underlying features (Figure 1C).

### 2.3. Detection of Early Metabolic Marker Patterns for Glioma

**Figure 2:**
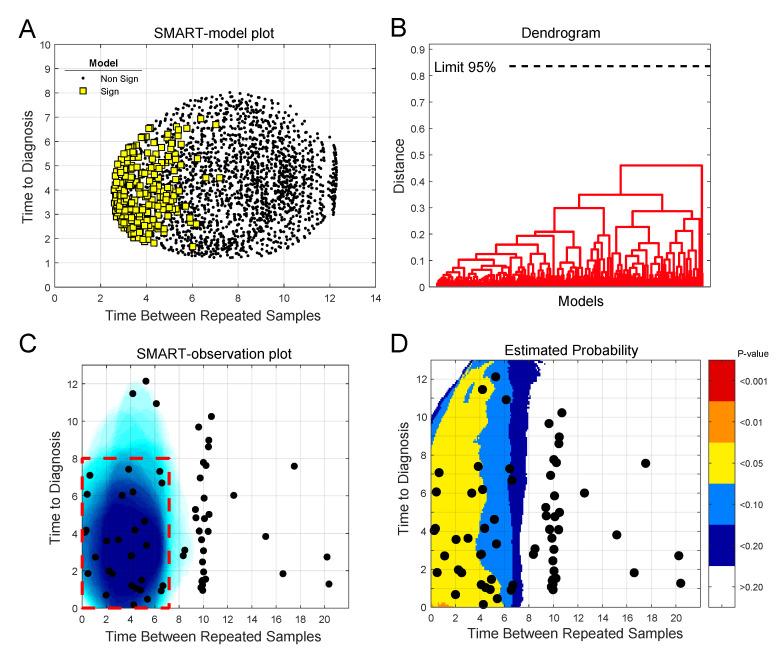
Use of the SMART procedure to find regions of interest for metabolic marker pattern detection for glioma. Subsets were selected by scanning time to diagnosis and time between repeated samples in steps of 0.25 years. In total, 2170 unique subsets, each consisting of twenty case-control observations, were created. (**A**) SMART-model plot showing mean time to diagnosis and time between repeated samples for the 2170 OPLS-EP models, out of which 277 models were statistically significant, *P* < 0.05 (yellow squares). (**B**) Dendrogram showing similarities between significant OPLS-EP models. All significant models had a similar underlying metabolic pattern, detected as one cluster. (**C**) SMART-observation plot for all glioma case-control observations showing the distribution pattern of subsets with significant OPLS-EP models (blue). Significant subsets were mainly composed of case-control observations collected less than eight years prior to diagnosis and with less than seven years between baseline and repeated samples, which defines the region of interest for detection of a glioma-related metabolic marker pattern (red dashed line square). (**D**) Estimated probability plot in comparison to simulated random data. The plot shows the probability (*P*-value) of obtaining the distribution pattern shown in (**C**) as a random event. Areas are color coded according to *p*-values < 0.001 to < 0.2, indicating the risk of a false positive result.

When we apply the SMART procedure on our glioma case-control data, consisting of 142 quantified metabolites for each sample, we see that the majority of the significant models are located to the lower left side in the SMART-model plot (Figure 2A). The plot indicates that subsets based on samples collected closer to diagnosis and with shorter follow up time between baseline and repeated samples more often give significant models. To describe each significant model, we examined their similarity by hierarchical cluster analysis (Figure 2B). We used loadings w to describe each model and defined the number of clusters using a 95% confidence limit. We see that all significant models group into one single cluster, indicating that all significant models builds on the same or a very similar underlying metabolic marker pattern. The vast majority of significant models were based on case-control observations collected less than eight years before diagnosis and with less than seven years between baseline and repeated samples, as seen in the generated SMART-observation plot (Figure 2C, blue shading). This time span defines the region of interest for metabolic marker pattern detection (Figure 2C, red dashed line square).

A fear when fitting many models on subsets of the data is that one may overfit the data in search of a significant *P*-value. To determine the validity of the obtained models we conducted simulations using random data, instead of glioma case-control progression data. The results of 10,000 simulations show that there is a very low probability that the number of significant models obtained for the metabolomics data, in the region of interest for molecular marker pattern detection, occurs by chance (Figure 2D). The SMART-observations plot with estimated probability, comparing simulated random data to the real glioma metabolomics data, shows that the probability of defining this region by chance is below alpha 0.05. Our comparison to random data is in this case also a very stringent approach, as clusters of significant OPLS-EP models with similar underlying features are not obtained for the random data.

### 2.4. Analysis of Progression Patterns for Individual Metabolites

By breaking the SMART analysis down to individual metabolites, we can study each metabolite significance pattern in relation to the region of interest for metabolic marker detection, as defined in Figure 2C. Within this region, we find 15 statistically significant metabolites (Table 2).

To determine the ability for these 15 metabolites to separate controls from future cases, one can calculate the optimized predictive ability of these metabolites in the region of interest for disease detection. These calculations were restricted to a one-component OPLS-EP model to limit overfitting. Analyzing all case-control observation in the region resulted in a good model with a predict ability Q^2^-value of 0.59 and a CV-ANOVA *P*-value of 5.0 × 10^−5^. The vast majority of all future cases were diagnosed with high-grade, WHO III-IV glioma (Table S1). Restricting the analysis to include only high-grade glioma, constituting 79% of all case-control observations, resulted in a slightly improved Q^2^-value of 0.62 and *P*-value of 4.0 × 10^−5^. Narrowing down the analysis to include only future glioblastoma WHO IV cases, gave a similar predictive Q^2^-value of 0.61, but with a higher *P*-value of 0.0015, as the number of included observations decline (55% of all case-control observations).

**Figure 3:**
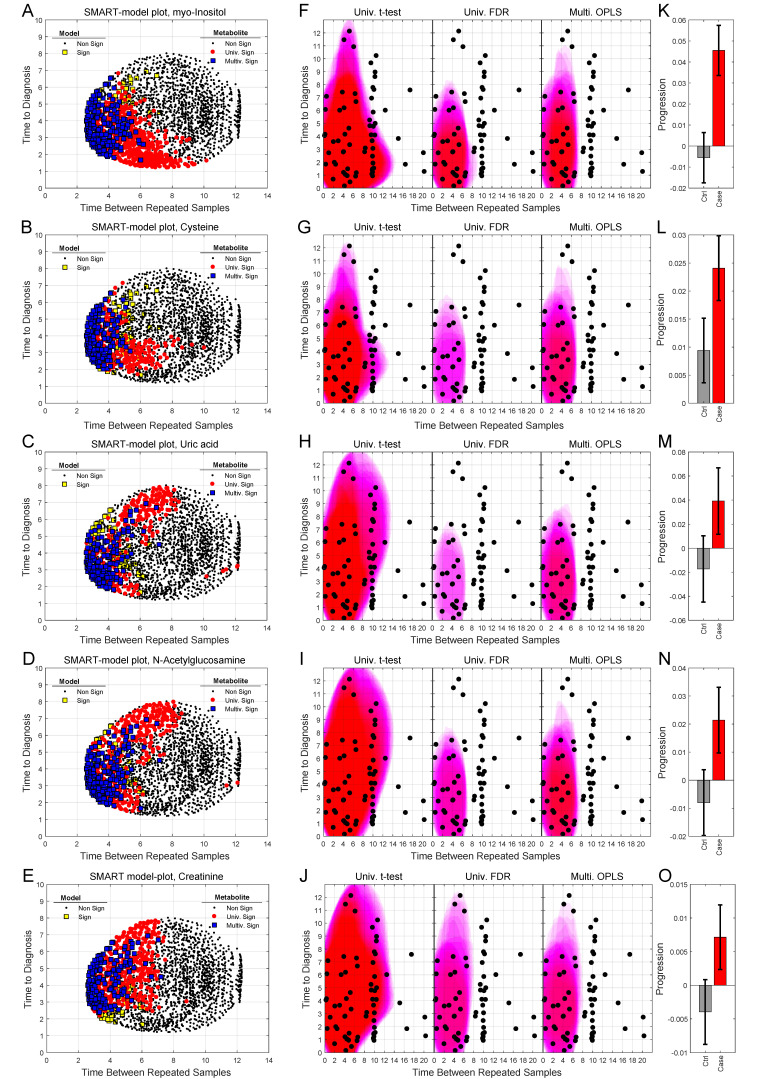
SMART-model and SMART-observation plots, and progression patterns for individual metabolites: myo-inositol (**A**,**F**,**K**), cysteine (**B**,**G**,**L**), uric acid (**C**,**H**,**M**), N-acetylglucosamine (**D**,**I**,**N**) and creatinine (**E**,**J**,**O**). (**A**–**E**) SMART-model plot for individual metabolites. The shape and color of the symbols relate to the statistical significance. For model: black dot; non-significant model, yellow square; significant model. For individual metabolite: black dot; non-significant metabolite, red dot; significant metabolite in univariate *t*-test, blue square; multivariate significant metabolite (w and p). (**F**–**J**) SMART-observation plots for individual metabolites. The SMART-observations plot is similar to the plot in Figure 2C but highlights the distribution pattern for subsets where the individual metabolites are significant, instead of models. Distribution pattern for three significance criteria are illustrated. Left panel: univariate *t*-test. Middle panel: univariate *t*-test with correction for Benjamini–Hochberg false discovery rate (FDR) < 0.2. Right panel: multivariate significance using loadings w and p. Distribution patterns shown in red color indicate significantly increased levels of the individual metabolite in cases compared to controls, while patterns shown in blue indicate significantly reduced levels of the metabolite in cases compared to controls. (**K**–**O**) Progression pattern for individual metabolites. Mean progression of metabolite levels for cases and controls in the defined region of interest for glioma. Confidence intervals (95%) based on a dependent two-sided *t*-test.

**Figure 4:**
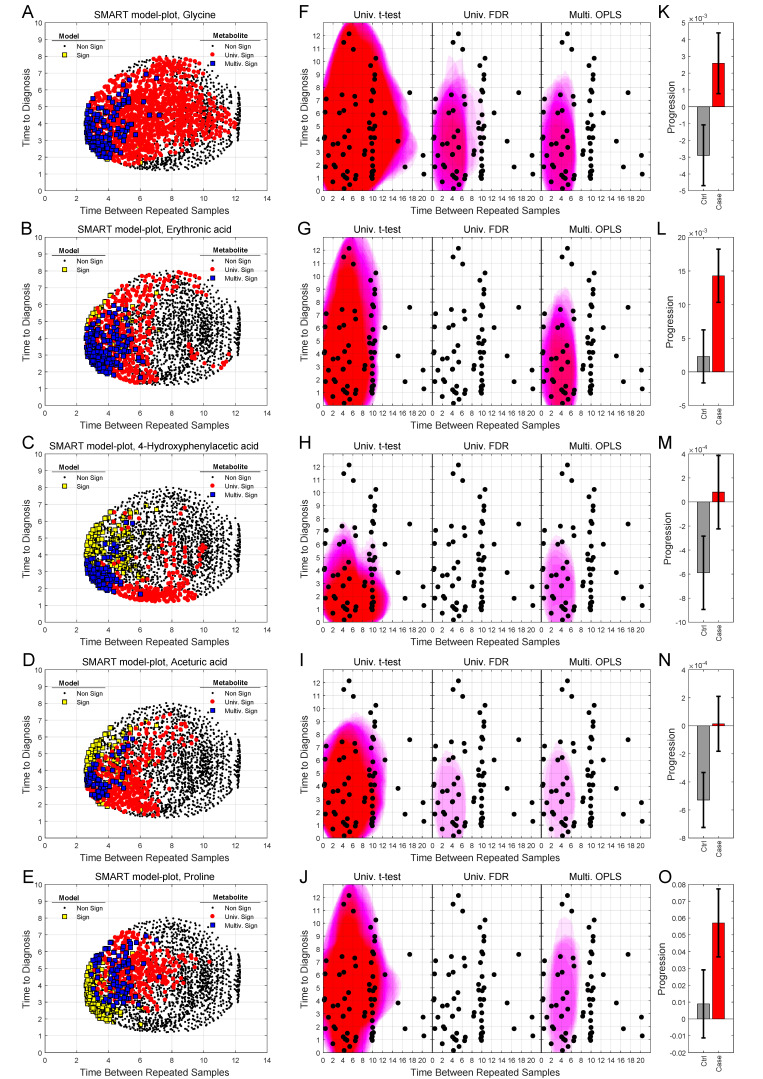
SMART-model and SMART-observation plots, and progression patterns for individual metabolites: glycine (**A**,**F**,**K**), erythronic acid (**B**,**G**,**L**), 4-hydroxyphenylacetic acid (**C**,**H**,**M**), aceturic acid (**D**,**I**,**N**) and proline (**E**,**J**,**O**). (**A**–**O**) As described in figure legend 3.

**Figure 5:**
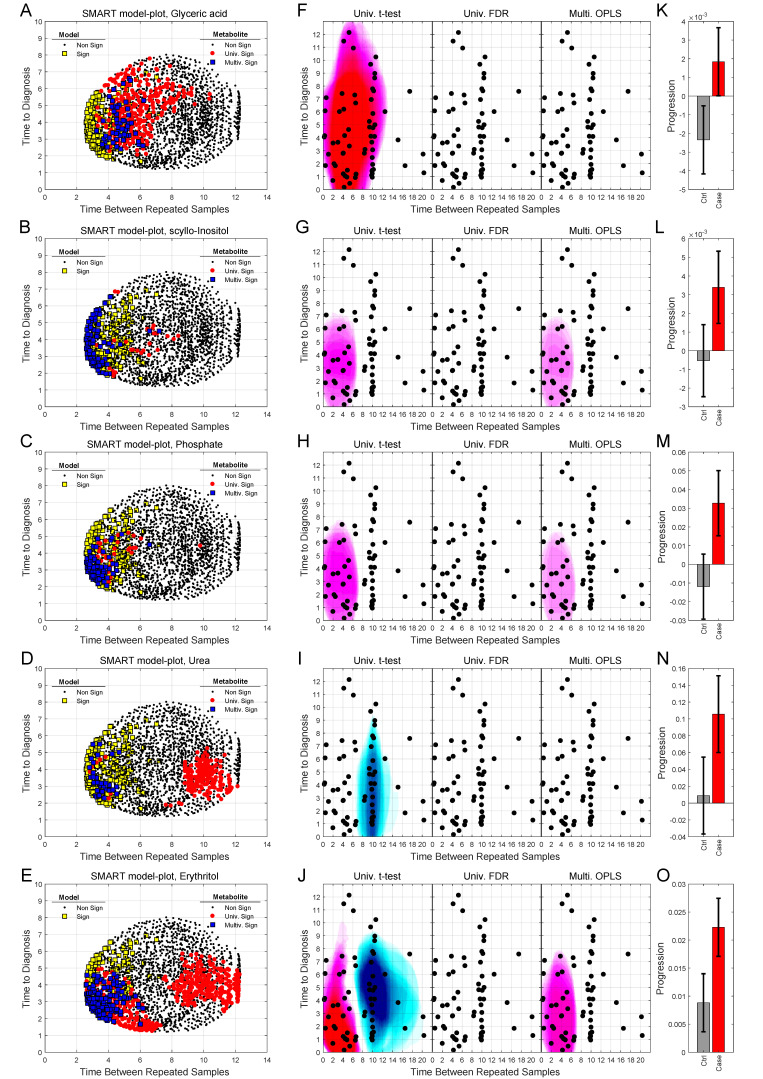
SMART-model and SMART-observation plots, and progression patterns for individual metabolites: glyceric acid (**A**,**F**,**K**), scyllo-inositol (**B**,**G**,**L**), phosphate (**C**,**H**,**M**), urea (**D**,**I**,**N**) and erythritol (**E**,**J**,**O**). (**A**–**O**) As described in figure legend 3.

We can also use SMART-model and SMART-observations plots to highlight the distribution of each individual metabolite, their statistical significance, and abundance for cases in comparison to controls. SMART-model plots, SMART-observations plots and progression patterns for all metabolites listed in Table 2 are shown in Figure 3, Figure 4 and Figure 5. We see statistically significant higher levels of myo-inositol, cysteine, uric acid, N-acetylglucosamine and creatinine, five compounds representing separate groups of interlinked metabolites, closer to diagnosis in the future glioma case compared to controls (Figure 3A–O). By evaluating these plots, we observe that significant metabolite levels correspond very well with significant OPLS-EP models (Figure 3A–E, yellow squares), when using the criteria for multivariate significance (w and p) (Figure 3A–E, blue squares), while univariate significant (*t*-test) metabolite levels highlight models spanning a much wider time span (Figure 3A–E, red dots) also including non-significant OPLS-EP models (Figure 3A–E, black dots). A very similar multivariate pattern can be observed for glycine and erythronic acid (Figure 4A–B, blue squares). The remaining eight metabolites show similar patterns but are multivariate significant in fewer models, in a narrower time frame appearing somewhat closer to diagnosis (Figure 4C–E and Figure 5A–E). The multivariate model significance criteria (w and p) strongly restrict the region of interest for metabolic marker detection, where individual metabolites are found to be significant (right panels in Figure 3F–J, Figure 4F–J and Figure 5F–J), in comparison to a univariate significance *t*-test (left panels in Figure 3F–J, Figure 4F–J and Figure 5F–J), and coincide in most cases well with a univariate *t*-test *P*-value corrected for using the Benjamini–Hochberg false discovery rate at 0.2 (middle panels in Figure 3F–J, Figure 4F–J and Figure 5F–J). Out of the 15 identified metabolites, seven metabolites including myo-inositol, cysteine, uric acid, N-acetylglucosamine, creatinine, glycine and erythronic acid (Figure 3F–J and Figure 4F–G) show the strongest multivariate significant pattern, appearing early, and consistently already eight years before diagnosis. Interestingly, all 15 metabolites were observed to increase in the plasma samples from glioma cases. The progression pattern within the defined region of interest for metabolic marker detection, shows clearly that the metabolite concentration increases in the plasma samples of glioma cases while unaffected in most matched controls (Figure 3K–O, Figure 4K–O and Figure 5K–O).

As a validation of the statistical significances for the 15 individual metabolites, we also here compared the glioma metabolomics data to random data (Figure S1). SMART-observation plots showing estimated probabilities for the individual metabolites after normalization to random data confirm similar distribution patterns for the individual metabolites as previously shown in Figure 3, Figure 4 and Figure 5.

### 2.5. Validation of the Identified Latent Biomarker for Glioma

**Figure 6:**
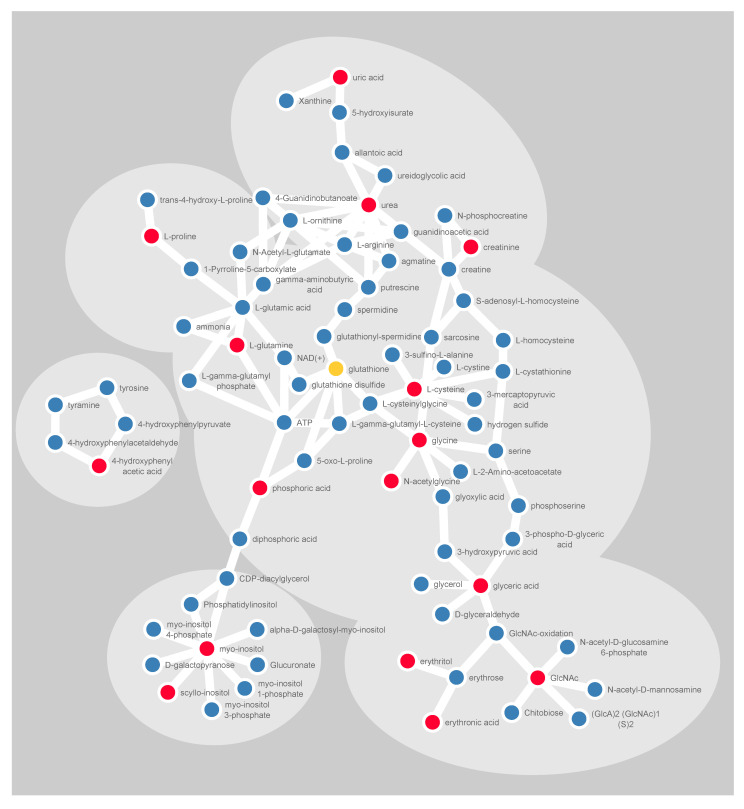
Schematic illustration of significantly altered metabolites (red) and their closest derivatives and precursors (blue). Glutathione is included in yellow, being a central metabolite in redox balancing. The linkage in the illustration is based on the KEGG pathway database, for humans only. Cytoscape software was used for visualizing molecular interactions.

A latent biomarker can be described as a systematic pattern of co-varying variables correlated to the phenotypic variation of interest. By use of SMART, we identify a latent biomarker for glioma, consisting of 15 significantly altered metabolites. Interestingly, almost all of these metabolites can be interlinked to each other through known human metabolic pathways (Figure 6). Either by being an upstream precursor molecule or a downstream derivative from known biochemical reactions, as described in the KEGG database. Detection of cohesive metabolites strengthens in our view the validity of the identified metabolic pattern. 

Nevertheless, to validate the identified latent biomarker and the time point when it becomes detectable, we analyzed a second set of pre-diagnostic EDTA-plasma samples (Table S2). The validation set consisted of tightly matched case-control samples collected at a single pre-diagnostic time point and was collected at the same biobank using identical sampling and storage procedures, which is a prerequisite for minimizing pre-analytical sample variability, that otherwise can cause unpredictable directional effects. The validation set was comparable to the discovery set regarding participants’ age at sampling, mean time to diagnosis, BMI, smoking status, fasting status, and clinical glioma diagnosis’s, but had a lower proportion of females.

**Figure 7:**
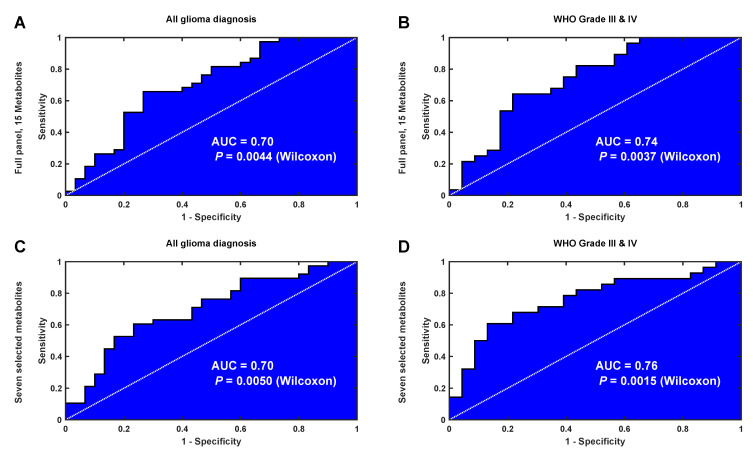
ROC curve analysis of the latent biomarker predicting glioma detection eight years before diagnosis in the single time point data set. (**A**) ROC curve for all glioma diagnoses using all 15 metabolites identified by SMART. (**B**) ROC curve for high-grade glioma diagnoses only using all 15 metabolites, as in (**A**). (**C**) ROC curve for all glioma diagnoses using a subset of seven metabolites identified with a strong and consistent significance pattern by SMART. (**D**) ROC curve for high-grade glioma diagnoses only using the same seven metabolites, as in (**C**).

For the validation set, we first calculated the differences between matched cases and controls (Equation (3)), followed by a calculation of the latent biomarker by use of OPLS. The output from the OPLS model highlights differences between matched cases and controls according to the latent biomarker, i.e., the panel of 15 metabolites, previously defined by SMART. Here, we observe a significant difference between matched cases and controls collected less than eight years before diagnosis (dependent *t*-test, *P* = 0.015, n = 38 pairs). The diagnostic ability for the latent biomarker can also be illustrated by use of receiver operating characteristic (ROC) curve analysis. Moreover, here, matched samples less than eight years before diagnosis significantly differ from samples collected more than eight years from diagnosis (Figure 7A, area under curve (AUC) = 0.70, Wilcoxon, *P* = 0.0044, n = 68 pairs). Although an AUC value of 0.70 does not describe a perfect separation, both results are significant and indicate that the identified latent biomarker contains valid information for disease detection. For the validation cohort, we also restricted the analysis to include only high-grade WHO III-IV glioma, constituting 75% of all case-control observations (Figure 7B). This sub analysis gave an improved AUC value of 0.74 and improved *P*-value of 0.0037. From the SMART plots of individual metabolites (Figure 3 and Figure 4), we observed seven metabolites, including myo-inositol, cysteine, uric acid, N-acetylglucosamine, creatinine, glycine and erythronic acid, with a particularly strong and consistent significance pattern. ROC analysis using only these seven metabolites resulted in a similar separation of cases and controls when analyzing all glioma diagnoses (Figure 7C, AUC = 0.70, Wilcoxon, *P* = 0.005, n = 68 pairs) but resulted in an improved AUC value of 0.76 and an improved *P*-value of 0.0015 when restricting the analysis to include only high-grade WHO III-IV glioma. Although our validation set was not optimal, containing only single time point samples, the identified metabolites can still separate future glioma cases from age and gender matched controls up to eight years before diagnosis.

X_difference_ = X_case_ − X_control_ (3)

## 3. Discussion

In this study, we introduce a modelling approach (SMART) for the unbiased definition of novel molecular marker patterns and boundaries for disease detection, using longitudinal case-control samples. Our study points out a metabolic marker pattern, or latent biomarker, detectable many years before glioma diagnosis. The latent biomarker consisting of 15 significantly altered metabolites includes many molecules with antioxidant properties, compounds related to oxidative metabolism or linked to monosaccharide metabolism and glutathione biosynthesis. Using SMART, we see a cohesive metabolite pattern with a traceable onset up to eight years before glioma diagnosis. Our data also indicate that metabolic marker patterns for glioma should optimally be based on repeated samples with a longitudinal follow up less than seven years apart. Our findings correlate well with a recent genetic study that investigated the genetic evolution of isocitrate dehydrogenase (IDH) wild-type glioblastoma using paired primary and recurrent tumor tissue. This study estimates the mutated founder cells with common genetic aberrations in the CDKN2A/B, PTEN, and EGFR loci to emerge two to seven years before diagnosis [6]. As indicated here, and in previous metabolic and genetic studies, glioma seems to have a much longer prodromal phase than previously anticipated.

Clinically, a blood test for glioma diagnostics could be relevant to discriminate unclear lesions at brain imaging or screening of high-risk individuals. In this study, we detect statistically significant and elevated levels of metabolites previously linked to tumor metabolic processes, which in our view strengthens the validity of the detected metabolic pattern. Our results are not to be interpreted as proofs that a diagnostic blood test for glioma is imminent. Affected metabolic and biochemical pathways are still to be characterized before clinical applications can be developed. Although these results should be interpreted with caution, the findings are still of great value for further studies using similar or complementary analysis approaches. The increase in myo-inositol concentrations were especially strong close to diagnosis. Inositol is a known antioxidant and several studies support myo-inositol as an important marker for glioma. A previous case-control study using single time point pre-diagnostic serum samples also found myo-inositol to be strongly associated with future glioblastoma development [7]. In incident cases of glioma, higher relative levels of myo-inositol in brain tumor tissue correlate with a less aggressive tumor progression, resulting in longer survival [8]. Earlier proton magnetic resonance spectroscopy studies showed reduced levels of myo-inositol in tumors of patients with more aggressive glioma phenotype, anaplastic astrocytomas and glioblastomas, compared to low-grade astrocytoma [9]. Myo-inositol levels have also been shown to increase in the extracellular compartment of the tumor during radiotherapy [10], while the contralateral normal-appearing white matter of un-treated glioblastoma patients has elevated myo-inositol levels, relative to age-matched healthy controls [11]. A recent magnetic resonance spectroscopy study also pointed out measurements of inositol/total choline ratio as the best discriminator between high- and low-grade glioma or brain metastasis [12]. Finally, a study comparing metabolites in patient-derived microdialysis fluids from high-grade tumor tissue to non-malignant brain tissue adjacent to the tumor pointed out elevated levels of myo-inositol in peripheral tissue surrounding the tumor [13]. The study also reported four- to eight-fold higher levels of glycine, proline and uric acid, and three-fold lower erythritol levels, in tumor microdialysate in comparison to levels in the adjacent non-tumoural brain tissue [13]. Intracellular myo-inositol forms phosphatidylinositol or myo-inositol-phosphates of various forms. However, myo-inositol does also maintain the osmotic balance between the tissue and its surroundings and protect cells from the negative impact of hyperosmolality [14]. The inositol accumulated following hypertonicity is transported into the cells rather than synthesized. A plausible explanation to the altered levels of myo-inositol in high- and low-grade tumors, the extracellular compartment and in blood is that myo-inositol levels are altered because of changed cellular monosaccharide metabolism, as high glucose uptake is a hallmark of tumor cells. Previous metabolic studies showed that intracellular myo-inositol levels are dependent on glucose concentration and that intracellular myo-inositol is depleted under high glucose conditions, in a competitive, dose-dependent manner [15,16,17].

The majority of the altered metabolites found in this study have previously been linked to redox balancing and brain tumor development [7,13,18]. Reactive oxygen species (ROS) are constantly produced and removed under physiological conditions. Oxidative damage including DNA mutation and epigenetic changes can contribute to malignant transformation. Glutathione, a tripeptide synthesized intracellularly from cysteine, glycine and glutamic acid, constitutes the core of the non-enzymatic intracellular antioxidant system. Glutathione redox balancing works in tandem with major extracellular antioxidants including uric acid, ascorbate, tocopherols, carotenes, and extrinsic bioflavonoids [18]. Our study points out elevated levels of cysteine, glycine, glyceric acid, aceturic acid (N-acetylglycine) and phosphate (phosphoric acid), mainly inter-linked through glycine-serine-threonine metabolism as well as directly linked to glutathione biosynthesis through the methionine metabolic pathway. Elevated serum levels of cystine, the oxidized form of cysteine, has previously been reported in future glioblastoma cases [7]. Our study points out higher plasma levels of 4-hydroxyphenylacetic acid, a colonic metabolite with potential cyclooxygenase (COX) inhibitory function and free radical scavenging properties, as well as proline, another principal organic osmolyte in brain [14]. Proline degradation is catalyzed by proline oxidase (also known as proline dehydrogenase) and is widely expressed in brain and other tissues. Proline oxidase is a hotspot for mutations and putative tumor suppressor, and decreased proline oxidase activity has been reported in glioblastoma tissue compared to normal tissue [19]. Analysis in mouse models has shown that pathways involved in glutathione metabolism, and the amino acids involved in glutathione biosynthesis, are the most significantly upregulated pathways during tumor initiation, and progressively increase to meet the demands of tumorigenesis [20].

Erythronic acid, erythritol and N-acetylglucosamine (GlcNAc) belong to another group of significantly altered compounds. Erythritol has excellent hydroxyl radical scavenger properties [21]. The reaction of erythritol with hydroxyl radicals results in the formation of erythrose that can be oxidized to form erythronic acid. Both erythrose, erythronic acid and glyceric acid have been identified as products when N-acetylglucosamine is oxidized by NaOCl [22], that relates to ROS degradation of connective tissue [23,24]. Creatinine, urea and uric acid belong to the last group of interlinked metabolites found in this study. Creatinine, the breakdown product of creatine-phosphate, originate mainly from muscle metabolism, while urea is the main metabolic end product of protein catabolism. Increased blood urea, serum creatinine, and N-acetylglucosaminidase have been reported in rats upon oxidative damage [25]. Uric acid is a potent antioxidant and the metabolic end-product of purine degradation by xanthine oxidase. Xanthine oxidase catalyzes the transformation of hypoxanthine to xanthine and xanthine to uric acid, but also generates oxygen radical species (H_2_O_2_ and O^2−^) upon conversion, which can be harmful for tissues with high enzyme activity [26,27]. Xantine oxidase activity is shown to increase inflammatory responses [28], and significantly higher xanthine oxidase levels have been reported in brain tumor tissues [29]. Uric acid is not an inert metabolite, since it acts as a pro- and antioxidant and activator of immune response and inflammation through COX-2 activation [27,28,30].

Inflammation is a plausible link between ROS and glioma development. Several epidemiological studies have investigated the relationship between long-term use of pro-inflammatory COX-1 and/or COX-2 inhibitors and glioma risk. The long duration of aspirin use reduces the risk of developing glioma by 30–50% in clinic-based cases and controls [31,32]. Mixed effects of aspirin/NSAIDs usage have been reported in other meta-studies, indicating large differences in study design [33,34]. Epidemiologic studies suggest inverse relations between glioma and long duration of diabetes, asthma and allergy, were anti-inflammatory medication is used [35,36,37]. These studies highlight the long-term protective trends and the need to specifically decipher the targeted anti-inflammatory mechanisms.

## 4. Materials and Methods

### 4.1. Study Subjects and Sample Acquisition

Plasma samples were obtained from the Northern Sweden Health and Disease Study (NSHDS), a prospective, population-based biobank consisting of blood and questionnaire data from primarily 40, 50 and 60-year-old individuals, collected in connection with health surveys or at time point for mammography screening. NSHDS blood samples were collected in the morning from fasting donors. Analyzed samples were collected in EDTA plasma vacutainers, frozen within 1 h and stored at −80 °C at the Biobank North at Umeå University Hospital. We included plasma samples collected from October 1986 to February 2010 from incident cases diagnosed with glioma after the sampling. We identified 132 individuals diagnosed with glioma. Out of these, 64 individuals had donated two pre-diagnostic blood samples and 68 had donated one pre-diagnostic sample. We also randomly selected 132 controls (cancer-free at inclusion) with single or repeated samples, carefully matched on age (± 5 months, mean 65 days), sample collection date (± 2 months, mean 32 days for both first and second collection time) and gender. The control group was also balanced for individual fasting time, BMI and sample thawing cycles (Tables S1 and S2). Most samples (379 out of 392) had not been thawed prior to this analysis. All samples were linked to the Swedish Cancer Register, an ongoing national cancer registry started in 1958 with 98 percent coverage of all cancer cases in Sweden. Median cancer-free follow up for included controls was 19.1 years. The precision that the included control individuals are cancer-free is therefore very high. Glioma cases were classified according to the International Classification of Diseases for Oncology, with glioblastoma representing the majority of all cases. Information on IDH 1/2 mutation status was not available, as all cases were diagnosed with glioma prior to implementation of the WHO2016 classification of brain tumors. None of the cases had prior history of cancer. The study group was homogeneous with respect to ethnicity resulting in a predominantly Caucasian northern Swedish cohort. All donors in the NSHDS cohort have given informed broad consent for the use of their samples in cancer research. This study was approved by the Regional Ethical Review Board at Umeå University, Umeå Sweden. Ethical approval number 2017-295-31M and 2018-87-32M.

### 4.2. Special Reagents

All chemicals were of analytical grade. The isotopically labeled internal standards [1,2,3-13C3]-myristic acid were purchased from Cambridge Isotope Laboratories (Andover, MA, USA), [2H6]-salicylic acid from Icon (Summit, NJ, USA) and [13C12]-sucrose was from Campro (Veenendaal, Netherlands). The stock solutions for internal standards were prepared in 0.5 μg/μL concentrations in methanol or water prior to metabolite extraction. Silylation-grade pyridine and N-Methyl-N-trimethylsilyltrifluoroacetamide with 1% trimethylchlorosilane were purchased from Restek (Bellefonte, PA, USA).

### 4.3. Metabolite Extraction and Analysis

The plasma samples were divided into analytical batches, preserving case-control interrelation and longitudinal sample pairs. Frozen aliquots of plasma were thawed on ice at room temperature. Metabolite extraction was performed using methanol:water extraction mix (90:10 *v*/*v*, including internal standards) and derivatized for GC–MS analysis as previously described [7]. The study samples were subjected to constrained randomization within the analytical batches [4]. In the analytical run, both longitudinal samples from the case and the corresponding control were consequently run in the same batch and directly adjacent to each other in random order, thereby minimizing variability in platform performance across matched pairs. The trimethylsilylated metabolites were analyzed with a Leco Pegasus HT time-of-flight mass spectrometer equipped with an Agilent 7890A gas chromatograph. Leco ChromaTOF software was used for instrument control and raw data acquisition. The column used for the GC separation was a 30 m, 0.25 mm inner diameter DB5-MS UI column with a 0.25 µm thick stationary phase. Splitless injection of 1 μL sample was performed with a PAL auto sampler system at an injection temperature of 270 °C. The purge time was 75 s with a rate of 20 mL/min. Helium was used as carrier gas with a flow rate of 1 mL/min. The primary GC oven temperature was held constant at 70 °C for 2 min and then ramped at 20 °C/min to 320 °C, where it was held constant for 8 min. The transfer line temperature between the gas chromatograph and mass spectrometer was set to 250 °C. Electron impact ionization at 70 eV was employed with an ion source temperature of 200 °C. Mass spectra were collected in the mass range of m/z 50 to 800 at 20 Hz and 1670 V detector voltage. A series of n-alkanes (C8–C40) were used as external retention index standards. As an additional quality control measure of analytical performance across and within samples batches, we analyzed a pooled plasma quality control reference sample at the beginning and end of each analytical batch, as well as between every 20^th^ study sample.

### 4.4. Metabolite Identification and Quantification

Acquired raw data were exported to MATLAB (Mathworks, Natick, MA, USA) in NetCDF format and processed using a curve resolution script, developed in house [38]. The procedure generates chromatographic profiles for each compound in each sample with a corresponding common spectral profile. We used the integrated area under the resolved chromatographic profile for quantification. The identity of the resolved peaks was determined by comparing mass spectra and retention indices with data in the Swedish Metabolomics Centre in-house GC–MS library. NIST MS search 2.3 software was used for manual verification of spectral identification. Compounds with a “spectral match score” below 700 and RI deviation larger than 25 units from the reference value were excluded. For identification with high confidence, all major fragment ions in the library hit should be present in the resolved spectra with a correct spectral intensity profile. Only compounds identified with high confidence and detected in all cases and control, in baseline and repeated samples, and in both study cohorts, in total 142 metabolites, were included in the statistical analysis. We did not detect D-2-hydroxyglutarate, a metabolite produced by IDH1/2 mutated cells, in these samples.

### 4.5. Multivariate Statistical Modeling

The data consisted of two independent sets of plasma samples: a baseline and repeated time points set consisting of 64 future cases and 64 individually matched controls, in total 256 samples, and a single time point set with 68 future cases and 68 individually matched controls, in total 136 samples (Tables S1 and S2). Orthogonal projections to latent structures (OPLS) were used for the modeling of differences between future cases and controls. We used both independent analysis OPLS-DA (discriminant analysis) and dependent analysis OPLS-EP (effect projections). For the OPLS-DA models, data were centered and scaled variable wise by subtraction of the mean intensity and division by the pooled standard deviation. For OPLS-EP models, data were not centered but variable wise scaled by division by the standard deviation. Both OPLS-DA and OPLS-EP were used in three different comparisons (Table 1): (i) for the baseline samples in the repeated time points set; (ii) for the repeated sample in the repeated time point set; (iii) for the progression pattern in the repeated time point set, where baseline data were subtracted from the repeated time point. The optimal OPLS models were achieved by a cross-validation strategy. In each round of cross-validation an OPLS model was calculated and only the variables (metabolites) reaching the significance criteria (loadings w and p (95%) in each round) were used for predictions of the response values for each sample in each separate cross-validation group. Q^2^ and *P*-values based on CV-ANOVA [39] were calculated based on the predicted responses.

We developed SMART to search for an optimal predictive area for variable selection—a region of interest for molecular marker detection—with respect to the variables’ time to diagnosis, and time between repeated samples. We scan the scatter plot using stepwise increments of 0.25 years in each direction. At each step, the twenty case-control observations with shortest time span to the selected time point were included. For the systematic scanning of the area surrounding the selected time point, the distances from the time point were calculated seven times using weights from 1/8 to 8 for the time to diagnosis variable, while the weight for time between repeated samples was kept constant at 1. The regions represented by the selected samples were estimated by a hoteling’s T2 ellipse (95%). In total, 2170 unique subsets were generated for the glioma data, each containing a combination of 20 out of the 64 case-control observations. For each subset an OPLS-EP model was calculated based upon case-control progression data and the model significance based on CV-ANOVA. A model was considered significant with a *P*-value CV-ANOVA < 0.05. To visualize contributing subsets, ellipses from the representative selections from significant models are stacked upon each other. This is visualized in images where the color intensity is related to the number of layers covering the different positions in the studied region (example in Figure 1A). From all significant models, the OPLS loadings (w) were stored. Similarities between the loadings were investigated using hierarchical cluster analysis, with 1-cosine similarity as distance measure and average linkage. The number of clusters were decided using a simulated 95% confidence interval. For individual metabolites, we describe the variable significance in the SMART-model plots by two statistical tests: (i) univariate significance *t*-test (*P*-value < 0.05); (ii) multivariate model significance using OPLS loadings w and p, which require a significant OPLS model and univariate significance (w) and significant cosine similarity (*P*-value < 0.05) between the variable and the response estimated by the model (p) [40]. Similar SMART-observation plots were created for individual metabolites, as for the models described above. Here, three different significance criteria were used: (i) univariate *t*-test (*P*-value < 0.05); (ii) univariate *t*-test (*P*-value < 0.05) applying Benjamini–Hochberg false discovery rate (FDR < 0.2) [41]; and (iii) multivariate model significance using the loadings w and p, as described above.

To estimate the significance for the region of interest, a simulation using random data was conducted. The SMART-observation plot, based on the 64 case-control observation, was recalculated 10,000 times using random number, from a population with mean zero and standard deviation one, instead of metabolite data. The probability of getting the same or higher intensities in the SMART-observation plot, when there are no real differences, was estimated by comparing the simulated data with the metabolite data. The probability is presented as six discrete levels: “>0.20”, “<0.20”, “<0.10”, “<0.05”, “<0.01” and “<0.001”.

The single time point cohort was predicted by an OPLS model from the repeated sample cohort, and the output form the OPLS model, the estimated latent biomarker, was evaluated using a dependent *t*-test and by ROC curve analysis. The significance of the ROC curve was estimated using The Wilcoxon signed-rank test. In the ROC curve analysis, the samples with less than eight years to diagnosis were seen as the positive condition.

### 4.6. Visualization of Molecular Networks

The linkage of significant metabolites and their closest derivatives and precursors was done based on publicly available information in the KEGG pathway database [42] for humans only. The interaction network was assembled by use of Cytoscape, an open source software platform for visualizing molecular interactions and biological pathways.

### 4.7. Data Accessibility

The SMART tool and data reproducing the results have been made accessible at Code Ocean https://doi.org/10.24433/CO.2960444.v2.

## 5. Conclusions

The presented SMART strategy facilitates molecular marker discovery and defines boundaries for early disease detection. This strategy consists of an analytical workflow, tailored to increase the statistical sensitivity for studies containing matched samples at repeated time points. This proof-of-concept study focuses on finding pre-diagnostic metabolic marker patterns for glioma. The SMART approach is, however, suitable for other types of molecular marker data, comprising sample sets with tightly matched and repeat samples. Here, we detect metabolic changes in blood plasma up to eight years before glioma diagnosis and discuss how the pattern of significant metabolites relates to redox metabolic pathways and to previously described tumor metabolic processes.

## Figures and Tables

**Table 1:** Summary of OPLS-DA and OPLS-EP models based on metabolite data from longitudinally collected plasma samples from future glioma cases and their matched controls. The optimization done in each round of cross-validation was based upon multivariate significant variables w and p ^(a^.

Data	Baseline Time Point Only ^(b^		Repeated Time Point Only		Progression Pattern	
Model	OPLS-DA	OPLS-EP	OPLS-DA	OPLS-EP	OPLS-DA	OPLS-EP
Observations	128	64	128	64	128	64
Components	-	-	1	2	1	1
R_2_Y	-	-	0.12	0.3	0.12	0.34
Q^2^	-	-	−0.28	0.01	−0.12	0.11
*P*-value _(CV-ANOVA)_	-	-	1	0.96	1	0.026

**^(a^** OPLS loadings w and p. Multivariate significance require a significant OPLS model, and univariate significance (w, *P*-value < 0.05), and significant cosine similarity between the variable and the response estimated by the model (p, *P*-value < 0.05). **^(b^** No models were calculated for the baseline time point only since no significant variables were found in each cross-validation round.

**Table 2:** Summary of significant metabolites.

Metabolite	HMDB ID	OPLS Loadings w ^(a^		OPLS Loadings p ^(b^	
		*t*-Value	*P*-Value	*t*-Value	*P*-Value
myo-Inositol	HMDB0000211	4.37	0.0002	7.73	<0.0001
scyllo-Inositol	HMDB0006088	2.08	0.047	3.08	0.0047
Cysteine	HMDB0000574	2.61	0.014	2.94	0.0067
Glycine	HMDB0000123	3.10	0.0044	2.83	0.0087
Glyceric acid	HMDB0000139	2.35	0.026	2.61	0.015
Aceturic acid (N-acetylglycine)	HMDB0000532	2.84	0.0083	2.36	0.026
Phosphate (phosphoric acid)	HMDB0002142	2.62	0.014	4.26	0.0002
Proline	HMDB0000162	2.43	0.022	4.11	0.0003
4-Hydroxyphenylacetic acid	HMDB0000020	2.25	0.032	3.31	0.0027
Erythronic acid	HMDB0000613	3.11	0.0043	4.12	0.0003
Erythritol	HMDB0002994	2.66	0.013	3.70	0.001
N-acetylglucosamine (GlcNAc)	HMDB0000215	2.57	0.016	4.05	0.0004
Creatinine	HMDB0000562	2.37	0.025	2.37	0.025
Uric acid (urate)	HMDB0000289	2.09	0.046	3.20	0.0035
Urea	HMDB0000294	2.17	0.039	3.12	0.0043

^(a^ Equivalent to a paired two-tailed Students *t*-test. *P*-value calculated using 28 degrees of freedom, ^(b^ t-value for cosine similarity (cs) between metabolite and model estimated response. *P*-value calculated using 27 degrees of freedom. *t*-value calculated using the formula: t = (cs*√(n − 1)) / √(1 − (cs)^2^).

## Supplementary Materials

The following are available online at https://www.mdpi.com/2072-6694/12/11/3349/s1, Figure S1: Comparison of SMART-observation distribution pattern for individual metabolites to random data, Table S1: Characteristics of the study population with pre-diagnostic repeated blood samples, Table S2: Characteristics of the study population with pre-diagnostic single blood samples.
