# Supplementary material for: Identification of Pre-Diagnostic Metabolic Patterns for Glioma Using Subset Analysis of Matched Repeated Time Points
Source: Cancers (Basel). 2020 Nov 12;12(11):3349. doi: 10.3390/cancers12113349 (PMC7696703; doi:10.3390/cancers12113349)

Supplementary Figure S1

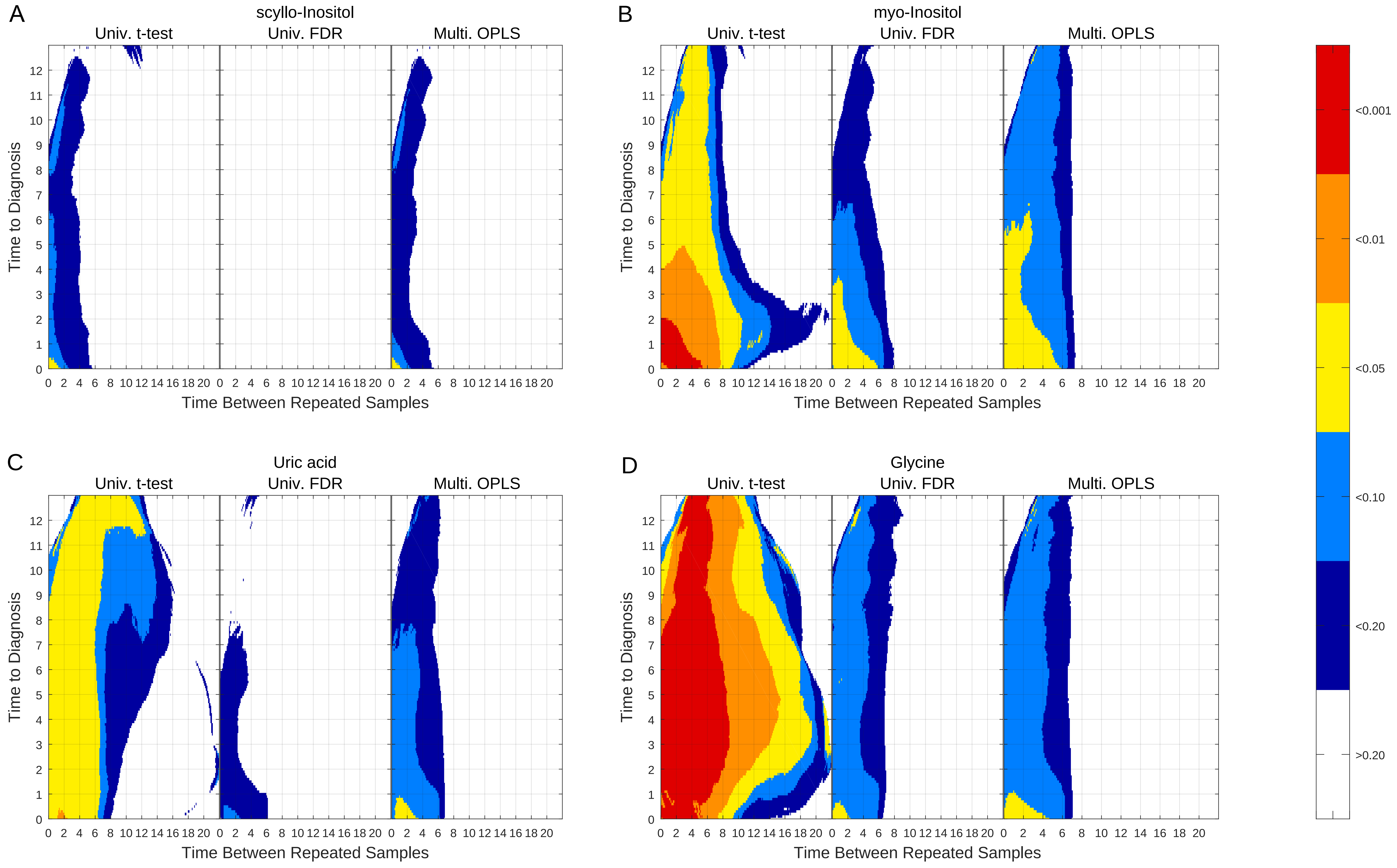

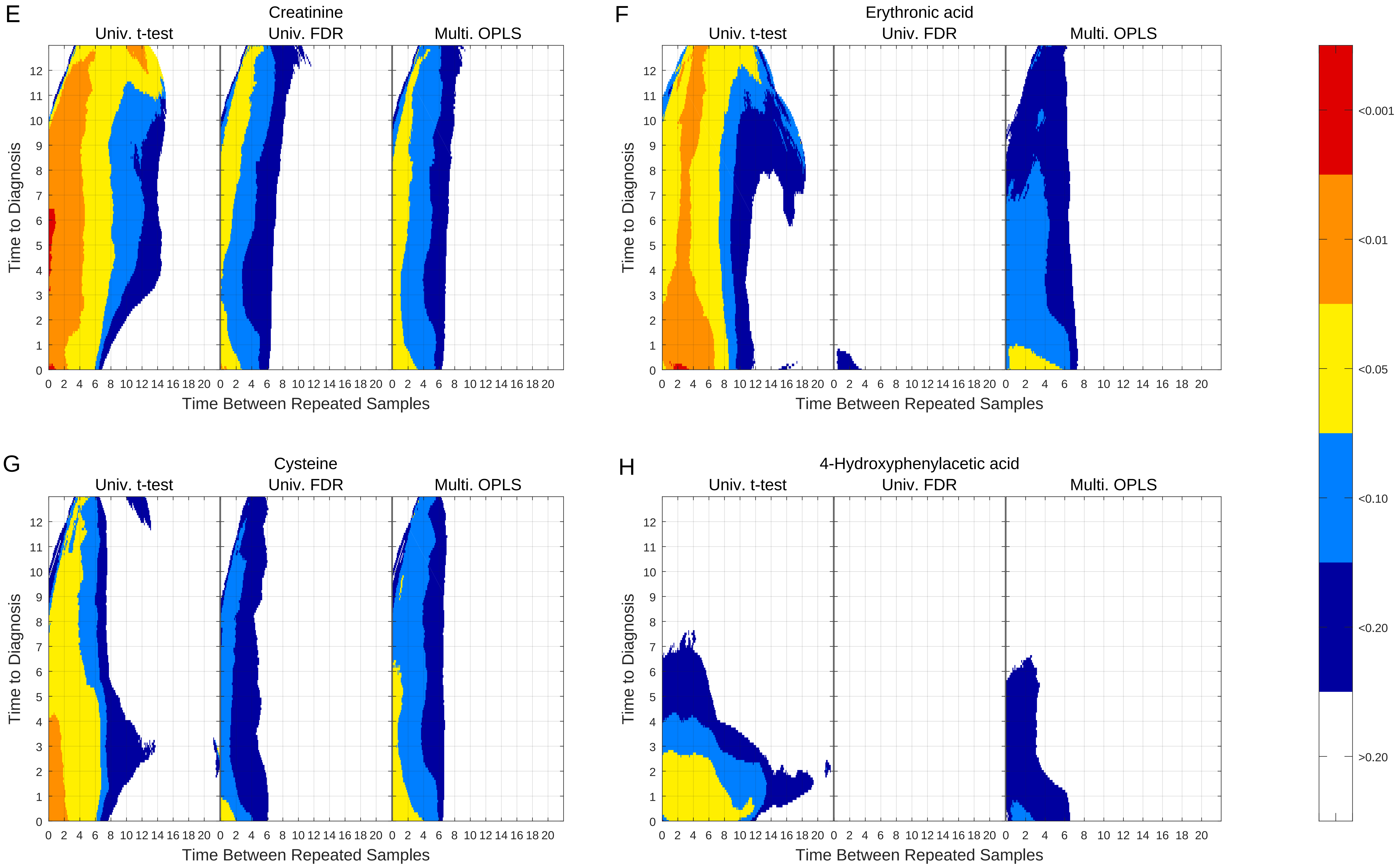

E
Creatinine
F
Erythronic acid
G
Cysteine
H
4-Hydroxyphenylacetic acid
Univ. t-test
Univ. FDR
Multi. OPLS
Time to Diagnosis
Time Between Repeated Samples
<0.001
<0.01
<0.05
<0.10
<0.20
>0.20

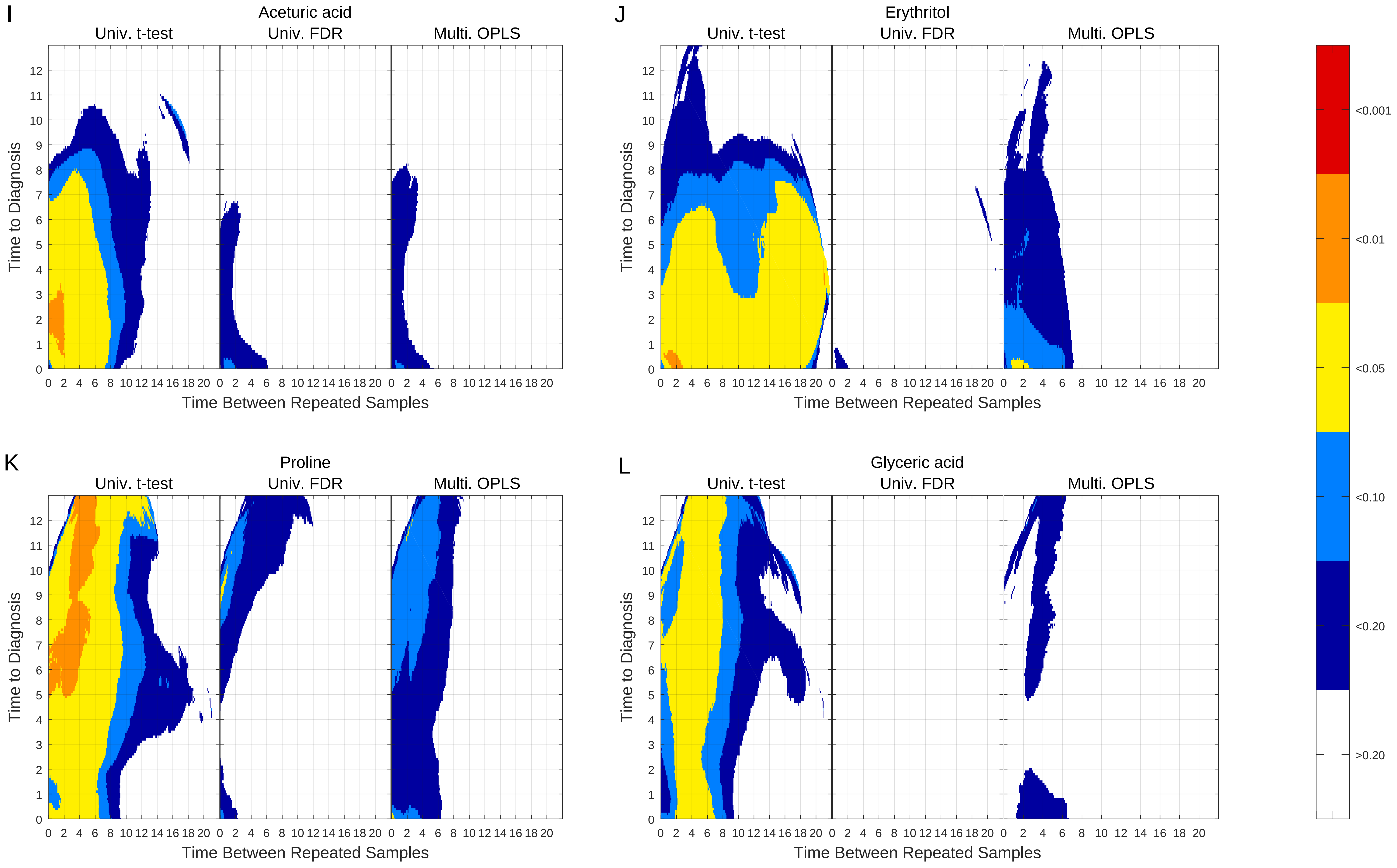
I
Aceturic acid
Univ. t-test
Univ. FDR
Multi. OPLS
J
Erythritol
K
Proline
L
Glyceric acid
Time to Diagnosis
Time Between Repeated Samples
0 2 4 6 8 10 12 14 16 18 20
0 1 2 3 4 5 6 7 8 9 10 11 12
<0.001
<0.01
<0.05
<0.10
<0.20
>0.20

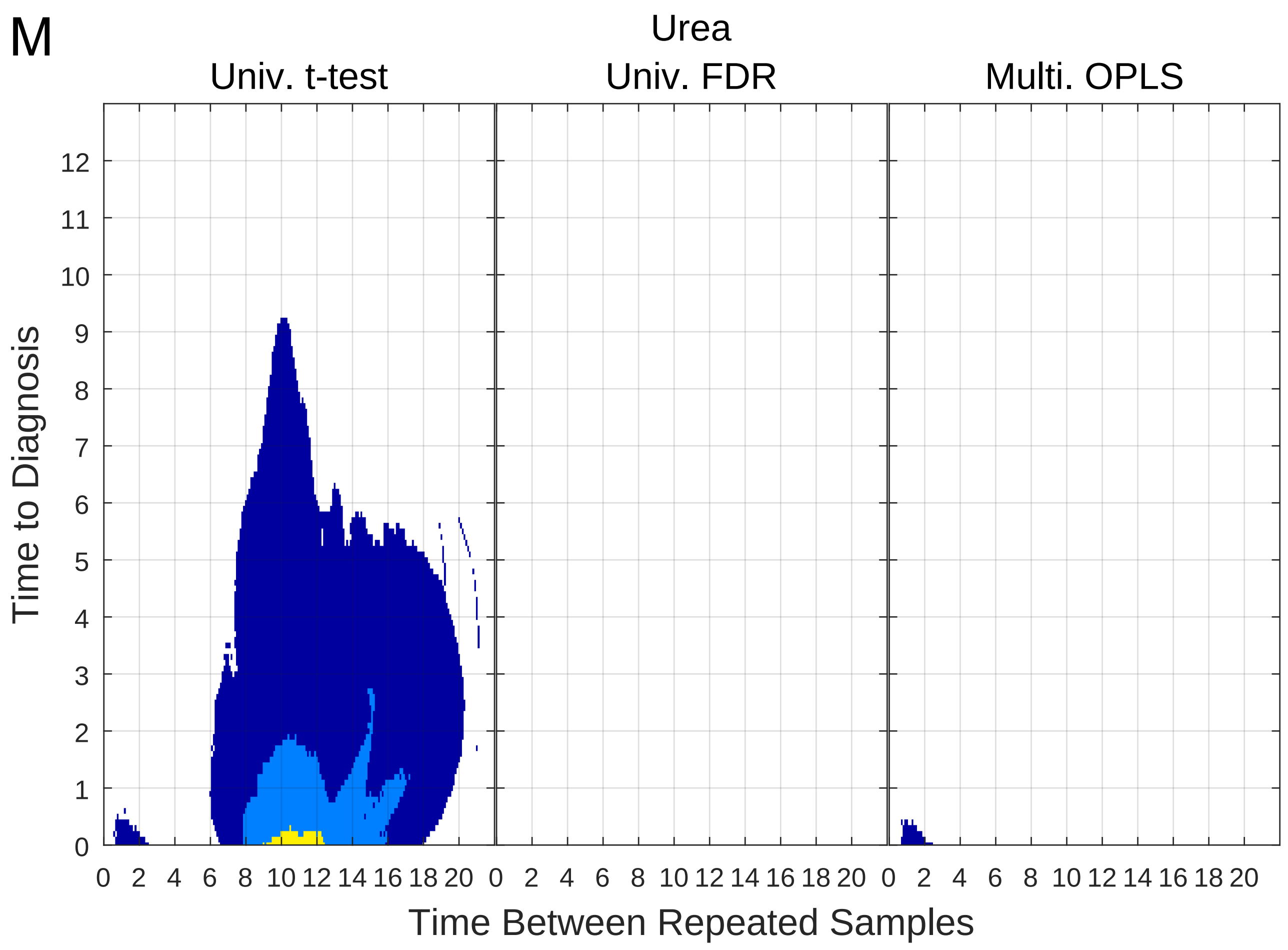

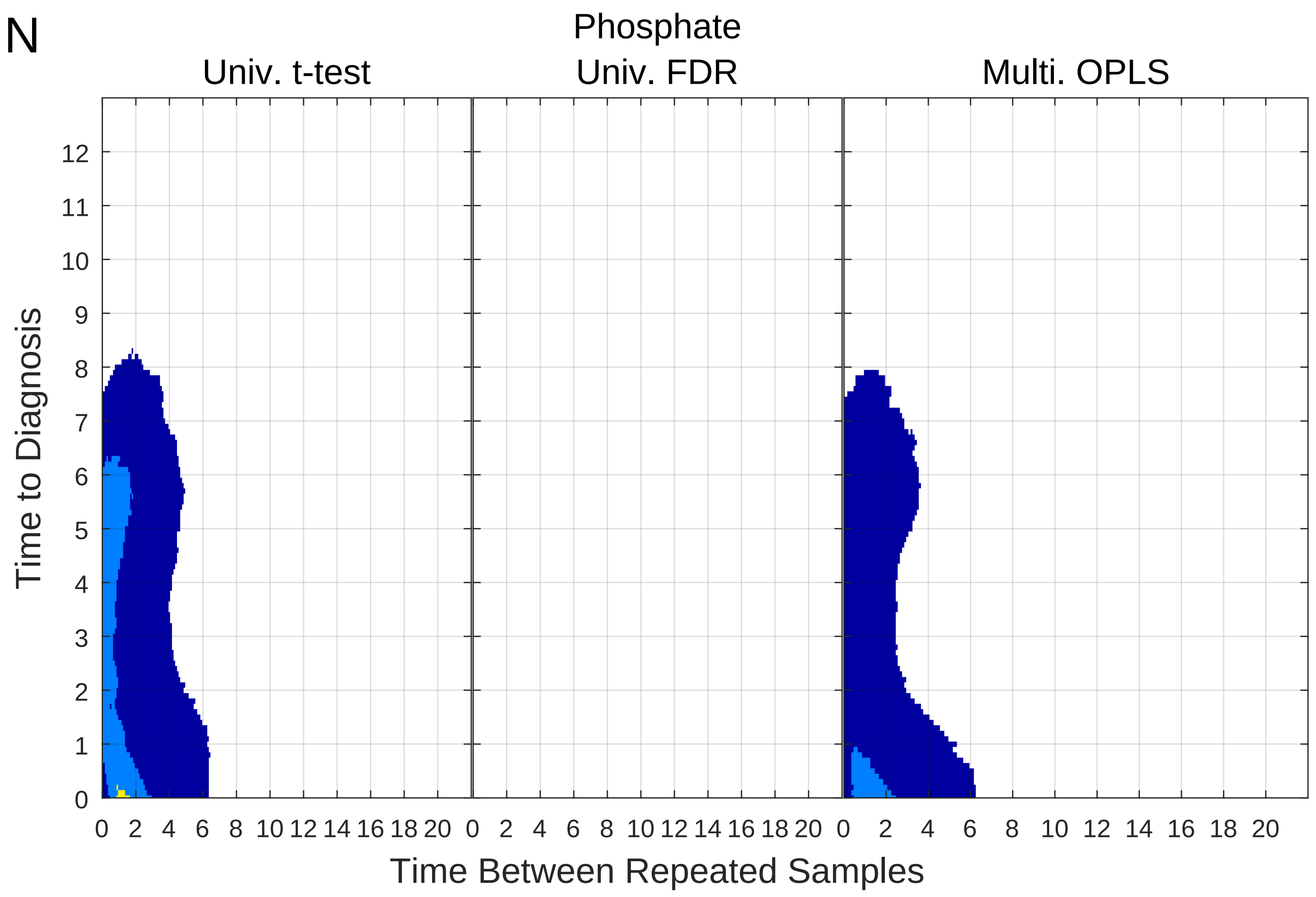

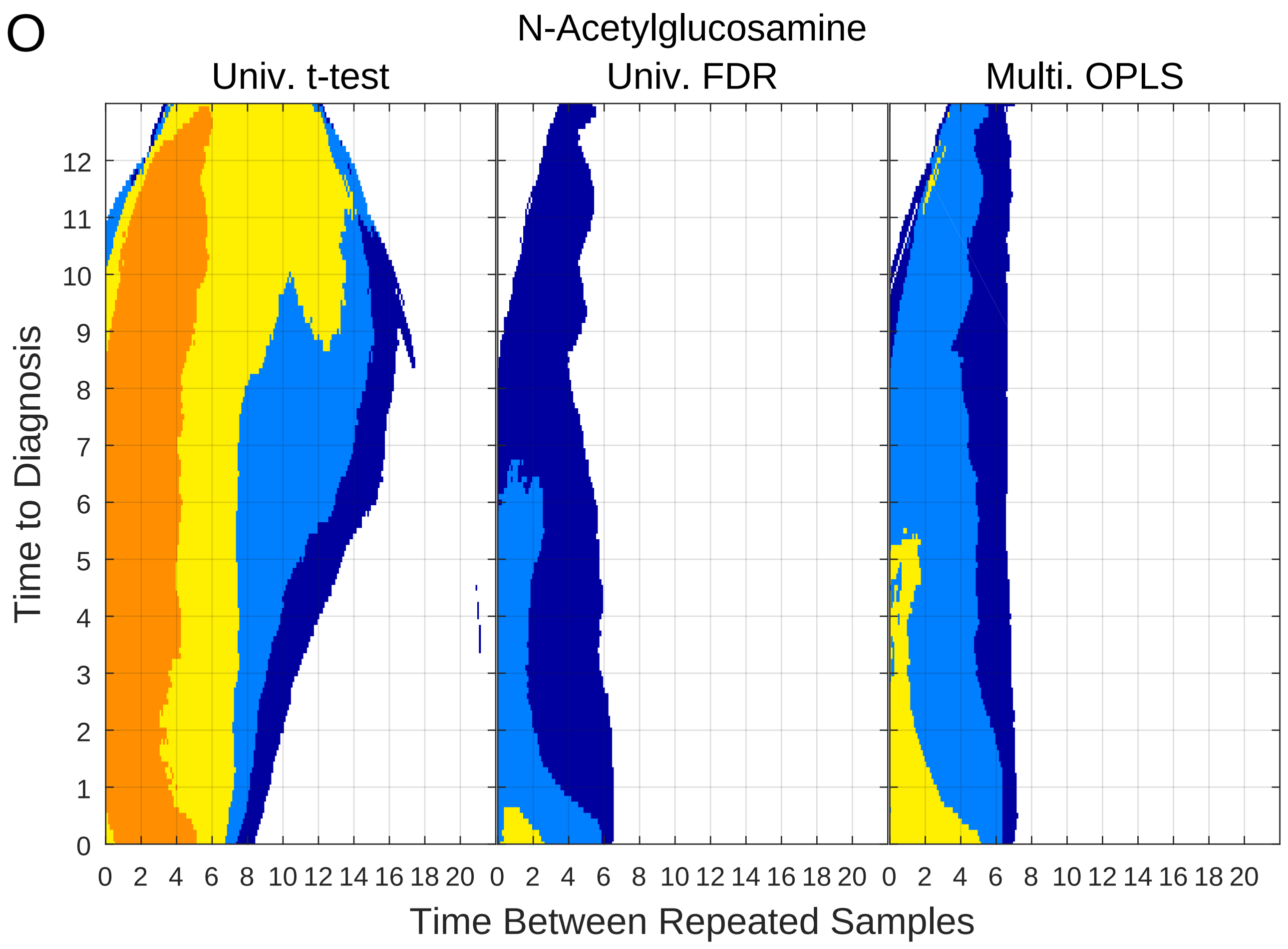

Supplementary Figure S1 description:
To validate the statistics for the 15 metabolites found to be significant in the region of interest for biomarker pattern detection, we compared their individual SMART-observation distribution pattern to simulated random data. (A-O) SMART-observation plots for the 15 metabolites highlighting estimated probability of the intensities when comparing the individual metabolite to the 95 percentile for the simulated data. An estimated probability of < 0.05 means that the probability of getting this results as a random event, is less than 5%. For each metabolite the estimated probability was shown for univariate t-test (left panel), univariate t-test with FDR < 0.2 (center panel) and using multivariate OPLS significance (right panel). The plots shows that the region of interest is also highlighted for individual metabolites.

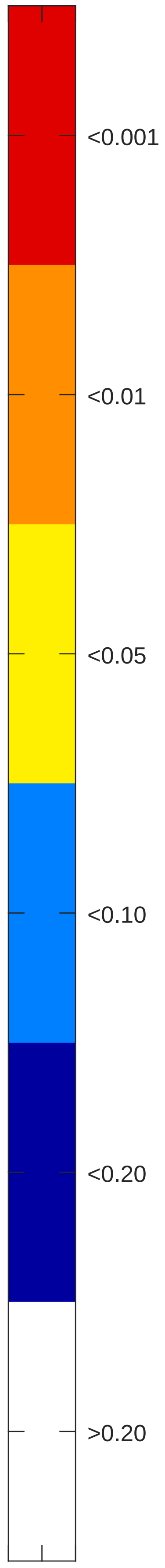

Supplement: Supplementary file 1 [file cancers-12-03349-s001.zip › Supplementary Figure S1.pdf]
