# Supplementary material for: Identification of Pre-Diagnostic Metabolic Patterns for Glioma Using Subset Analysis of Matched Repeated Time Points
Source: Cancers (Basel). 2020 Nov 12;12(11):3349. doi: 10.3390/cancers12113349 (PMC7696703; doi:10.3390/cancers12113349)
Supplement: Supplementary file 1 [file cancers-12-03349-s001.zip › Supplementary Table S1.pdf]

**Supplementary Table S1. Characteristics of the study population with pre-diagnostic repeated blood samples**

| | Cases | | Controls | | P-value[a] |
|---|---|---|---|---|---|
| | N | % | N | % | |
| | 64 | | 64 | | |
| Age at sample collection, years | | | | | b |
| Baseline sample, mean (range) | 48.8 | (30-61) | 48.8 | (30-60) | |
| Repeated sample, mean (range) | 56.3 | (40-68) | 56.3 | (40-68) | |
| Time between blood draw and diagnosis, years | | | | | |
| Baseline sample, mean (SD) | 11.7 | 5.6 | - | - | |
| Repeated sample, mean (SD) | 4.2 | 3 | - | - | |
| Sex | | | | | b |
| Female | 50 | 78.1 | 50 | 78.1 | |
| Male | 14 | 21.9 | 14 | 21.9 | |
| Body mass index | | | | | |
| Baseline sample, mean (SD) | 24.7 | (3.2) | 25.7 | (3.2) | 0.18 |
| Unknown | 1 | | 2 | | |
| Repeated sample, mean (SD) | 25.9 | (3.5) | 26.3 | (3.4) | 0.56 |
| Unknown | 6 | | 9 | | |
| Smoking status | | | | | |
| Baseline sample | | | | | 0.50 |
| Non-smoker | 26 | 40.6 | 22 | 34.4 | |
| Current smoker | 10 | 15.6 | 17 | 26.6 | |
| Former smoker | 13 | 20.3 | 11 | 17.2 | |
| Unknown | 15 | 23.4 | 14 | 21.9 | |
| Repeated sample | | | | | 0.30 |
| Non-smoker | 20 | 31.3 | 14 | 21.9 | |
| Current smoker | 6 | 9.4 | 6 | 9.4 | |
| Former smoker | 4 | 6.3 | 10 | 15.6 | |
| Unknown | 34 | 53.1 | 34 | 53.1 | |
| Individual fasting status | | | | | |
| Baseline sample | | | | | 0.94 |
| 0-4 hours | 13 | 20.3 | 14 | 21.9 | |
| 4-8 hours | 13 | 20.3 | 14 | 21.9 | |
| > 8 hours | 38 | 59.4 | 36 | 56.3 | |
| Repeated sample | | | | | 0.60 |
| 0-4 hours | 33 | 51.6 | 33 | 51.6 | |
| 4-8 hours | 1 | 1.6 | 0 | - | |
| > 8 hours | 30 | 46.9 | 31 | 48.4 | |
| Thawing cycles before | | | | | |
| Baseline sample | | | | | 1.00 |
| no | 59 | 92.2 | 60 | 93.8 | |
| once | 5 | 7.8 | 4 | 6.2 | |
| Repeated sample | | | | | - |
| no | 64 | 100 | 64 | 100 | |
| once | 0 | - | 0 | - | |
| Glioma subtypes | | | | | |
| Glioblastoma | 43 | 67.2 | | | |

| | | |
|---|---|---|
| Oligodendroglioma | 6 | 9.4 |
| Anaplastic oligodendroglioma | 2 | 3.1 |
| Astrocytoma, anaplastic type | 6 | 9.4 |
| Astrocytoma | 2 | 3.1 |
| Glioma, not otherwise specified | 5 | 7.8 |

[a]P-values calculated using paired t-test for continuous- and Chi-square test for categorical variables as well as the Fisher's exact test when appropriate

[b]Matching factors
