# Supplementary material for: Identification of Pre-Diagnostic Metabolic Patterns for Glioma Using Subset Analysis of Matched Repeated Time Points
Source: Cancers (Basel). 2020 Nov 12;12(11):3349. doi: 10.3390/cancers12113349 (PMC7696703; doi:10.3390/cancers12113349)
Supplement: Supplementary file 1 [file cancers-12-03349-s001.zip › Supplementary Table S2.pdf]

Supplementary Table S2. Characteristics of the study population with pre-diagnostic single blood samples

	Cases		Controls		P-value[a]
	N	%	N	%	
	68		68		
Age at sample collection, years					b
Mean (range)	52.7	(30-68)	52.7	(30-68)	
Time between blood draw and diagnosis, years					
Mean (SD)	8	5.4	-	-	
Sex					b
Female	19	27.9	19	27.9	
Male	49	72.1	49	72.1	
Body mass index					0.11
Mean (SD)	26.0	(3.7)	26.9	(4.1)	
Unknown	2		3		
Smoking status					0.50
Non-smoker	27	39.7	19	27.9	
Current smoker	10	14.7	13	19.1	
Former smoker	19	27.9	24	35.3	
Unknown	12	17.6	12	17.6	
Individual fasting status					0.87
0-4 hours	9	13.2	11	16.2	
4-8 hours	13	19.1	12	17.6	
> 8 hours	46	67.6	44	64.7	
Thawing cycles before					1.00
no	66	97.1	66	97.1	
once	2	2.9	2	2.9	
Glioma subtypes					
Glioblastoma	37	54.4			
Oligodendroglioma	3	4.4			
Anaplastic oligodendroglioma	3	4.4			
Astrocytoma, anaplastic type	11	16.2			
Astrocytoma	7	10.3			
Gliosarcoma	2	2.9			
Glioma, not otherwise specified	5	7.4			

[a]P-values calculated using paired t-test for continuous- and Chi-square test for categorical variables as well as the Fisher's exact test when appropriate

[b]Matching factors
